# De Wet Swanepoel: using digital technologies to improve access to hearing health

**DOI:** 10.2471/BLT.24.030424

**Published:** 2024-04-01

## Abstract

De Wet Swanepoel talks to Gary Humphreys about leveraging the power of digital health technologies to improve access to hearing health care.

*Q: How did you to come focus on audiology so early?*

A: Here in South Africa, training in audiology begins at the undergraduate degree level, so you're essentially required to commit early, which I was happy to do. I'd been interested in communication from a young age, having grown up in a household with a father who suffered from noise-induced hearing loss related to hunting. His hearing loss began mildly and, growing up, my siblings and I were used to him missing parts of conversations. It was even funny sometimes. But, as it progressed, we saw that there was a darker side to it, leading to social isolation and retreat from certain situations like family get-togethers. I came to see how hearing loss strikes at the core of what makes us human: communication.

*Q: How is hearing loss defined in simple terms?*

A: Normal hearing is defined as being able to hear sounds of around 20 decibels or better in both ears, which is about as much noise as a whisper. Someone is said to have hearing loss if they are not able to hear that. It’s estimated that around 1.5 billion people are currently living with hearing loss, which is about 1 in 5 people. Around 430 million of them have disabling hearing loss (a hearing threshold greater than 35 decibels). However, fewer than 1 in 5 people who require hearing care receive it, and these people are primarily living in low- and middle-income countries.

*Q: What are the main obstacles to accessing hearing health services in South Africa?*

A: One is the severe shortage of hearing health-care professionals. This reflects a broader reality on the continent where it is estimated that there is less than one audiologist for every million people. At the same time, in South Africa, as elsewhere, the traditional hearing health service delivery model is centralized, reliant on expensive equipment and specialized personnel, making decentralization and scaling challenging.

*Q: When did you begin to focus on digital health solutions as a way of improving access?*

A: Around 2010, I was serving on a working group for the National Department of Health, and we were deliberating on how to implement a national strategy for universal vision, hearing and nutrition screening in South African schools. It was going to amount to millions of screenings annually and, as we were talking, it became apparent to me that the existing technologies, along with their operational requirements and associated costs, were not going to be sufficient. At that point digital technology was already bringing about significant changes in agriculture, banking and health care, and felt like it held similar promise for hearing services.

I was watching the way mobile phone providers were expanding connectivity across the continent, despite the up-front costs and limited consumer base. They clearly recognized that the technology was going to spread and rapidly reach the lower tiers of the socioeconomic pyramid. I became convinced that exploring smartphone-based technologies for simple screenings was the way forward. So, I teamed up with a colleague – Professor Herman Myburgh – who had a background in computer engineering, and we started working on a hearing screening device using the cheapest Android smartphone available. We ended up using a 20 United States dollar (US$) smartphone which we paired with some US$ 40 headphones and, after a lot of calibration and software adjustments, we achieved a screening process comparable to the gold standard pure tone audiology devices that are typically used in clinics, schools, and community-based programmes. We were even able to monitor background or environmental noise in real time, which was vital to achieving an accurate screening test in community settings.

*Q: You were able to replicate the performance of high-end audiometers costing tens of thousands of dollars?*

A: That’s right, but it’s important to note that we weren’t just looking to replicate. We felt that smartphones offered other advantages, including a user-friendly interface which was going to allow us to develop an accessible service delivery model. We basically wanted people with minimal training, but also limited literacy, to be able to operate the device and run a test. In the end, community health workers were able to do it. They didn’t need to know how to do audiometry; they just needed to know how to press a button to start, and how to indicate whether someone had responded or not. Everything else ran in the background using algorithms.

*Q: How is the test conducted?*

A: The recipient of the test is presented with a series of tones at different frequencies using an automated test sequence, and responds to what they hear by raising a hand or pressing a button. In the case of young children, facilitators assist in indicating the sound heard, while adults engage directly by pressing the button when they hear the tones. Using phones for testing also allowed us to digitize monitoring and surveillance. When we started out, the health ministry relied entirely on paper records, and no one really knew what was happening in the field – how many kids were screened, what their referral rates were, what were the follow-up rates. Being able to capture all the data through a smartphone and uploading these to a server made a huge difference.

*Q: Whose servers were you using and how did you ensure data security?*

A: We built our own system initially, and then worked with national partners to feed our data into an integrated health services portal for national health data. Of course, since then, these platforms have become much more stringent in terms of privacy requirements, and we have developed in line. But back then, we just had a portal. An individual’s response data would be encrypted and programme managers could remotely review the data, make recommendations and then send text messages through to the parents. That also closed a loop in the school screening programmes that were relying on letters that the child had to take back home and give to the parent to take action. With our system we were able to directly connect to the families, send the test result and the recommendation of where to go for a follow-up based on the geolocation information also derived from the phone.

*Q: But given the lack of specialists, didn’t they just hit another access wall?*

A: Indeed, they did, which was why we initiated the development of diagnostic test procedures for the same device. In 2016, we created a diagnostic version of the screening app called hearTest which used the same automated algorithm we’d developed for screening. This app made it possible to determine the degree of hearing loss, categorizing it as mild, moderate, or severe. Additionally, we embarked on an applied project to develop a smartphone-compatible otoscope (a medical device used by health-care professionals to examine the ear canal and the eardrum). We started by trying to attach a device to the smartphone camera itself and got it to work, but then every six months or so the phone model changed. So eventually, we worked with a manufacturer in south-east Asia to co-develop an otoscope that could be connected to any smartphone.

“Hearing loss strikes at the core of what makes us human.”

*Q: Were you able to capture and store images?*

A: Not only that, we used the captured images to train a decision-making algorithm that was able to diagnose pathologies such as otitis media, wax impaction, or conditions necessitating advanced medical attention. We launched the algorithm in 2016 and have continued to improve it. It now does a better job than general practitioners and matches the accuracy of ear, nose and throat specialists, achieving 94% accuracy in diagnosing common ear conditions. These diagnoses are then used to guide treatment recommendations, from the fitting of conventional hearing aids to referrals for specialized care at tertiary centres.

*Q: What impact have you had on improving access to hearing health treatment?*

A: Not as much as we would like, but we are working to change that also. Improving access to treatment has always been integral to our approach, and in 2021 we started providing hearing aids. This makes it possible for users to undergo a hearing test on their mobile phones and directly fit the device without clinic visits. We are currently working with the World Health Organization to trial decentralized service delivery models for hearing aids in low- and middle-income countries, particularly in low-income settings. In South Africa, we are leading a multi-centre study employing these technologies where community health workers conduct screenings, facilitate diagnostic testing, and provide hearing aids on the spot. The hearing aids are preset or can be programmed via smartphones. Despite these initiatives, many challenges remain across the continent where fewer than 3% of individuals in need of hearing aids receive them.

*Q: To what extent are digital hearing health technologies being taken up in the South African health system?*

A: Less than we’d like. The COVID-19 pandemic went some way to stimulating the uptake of digital applications for services such as audiology, and there was some relaxation of regulatory allowances which facilitated adoption, but there has since been a reduction in allowances, slowing momentum. So, we need to do more to make the case for digital hearing health.

*Q: And outside of South Africa?*

A: There are plenty of reasons for optimism. The social enterprise that we started with smartphone hearing screening now has about 180 employees, and a presence in South Africa and the USA. We also have partners around the world. To date, we have done hearing tests in more than 190 countries, benefiting more than 2 million patients, including in the USA. We developed our technology out of necessity in a context of resource poverty, but the same technologies can also boost the efficiency and effectiveness of service provision in better-resourced systems. Ensuring access to hearing health is a challenge for everyone, and it is only likely to get greater in the coming years, with a possible 700 million people living with disabling hearing loss requiring treatment by 2050. AI-augmented digital technologies of the kind we have been able to develop could go a long way to meeting demand.

